# MicroRNA-146a-5p attenuates irradiation-induced and LPS-induced hepatic stellate cell activation and hepatocyte apoptosis through inhibition of TLR4 pathway

**DOI:** 10.1038/s41419-017-0038-z

**Published:** 2018-01-18

**Authors:** Yuhan Chen, Zhifeng Wu, Baoying Yuan, Yinying Dong, Li Zhang, Zhaochong Zeng

**Affiliations:** Department of Radiation Oncology, Zhongshan Hospital, Fudan University, 180# Fenglin Road, 200032 Shanghai, China

## Abstract

Elevated toll-like receptor 4 (TLR4) expression is associated with a high risk of radiation-induced liver disease (RILD). MicroRNA (miR)-146a-5p is a key regulator of lipopolysaccharide (LPS)/TLR4 signaling, but its role in modulation of RILD remains unclear. Here, we found that irradiation and LPS stimulation induced TLR4 and miR-146a-5p expression in the human hepatic stellate cell (HSC) line LX2. Ectopic expression of miR-146a-5p in LX2 inhibited irradiation-induced and LPS-induced pro-inflammatory cytokine secretion and cell proliferation, and promoted cell apoptosis by down-regulating the expression levels of TLR4, interleukin-1 receptor associated kinase 1 (IRAK1), tumor necrosis factor receptor associated factor 6 (TRAF6) and phosphorylation of nuclear factor-kappa B. In addition, the culture medium from the irradiated and LPS-stimulated HSCs transfected with miR-146a-5p significantly attenuated apoptosis in irradiated hepatocytes. Overexpression of miR-146a-5p reduced α-smooth muscle actin production in irradiated and LPS-stimulated LX2 cells, which was associated with inhibition of TRAF6-mediated JNK and Smad2 phosphorylation. Knockdown of TRAF6 or IRAK1 mimicked the effects of miR-146a-5p on HSC function. Furthermore, miR-146a-5p treatment alleviated irradiation-induced and endotoxin-induced hepatic inflammatory response and fibrogenesis in mice through inhibition of the TLR4 signaling pathway. Collectively, this study reveals the anti-pro-inflammatory and anti-fibrotic effects of miR-146a-5p on liver injury, and suggests a potential application of miR-146a-5p in the therapeutic prevention of RILD.

## Introduction

Radiotherapy is one of the most effective treatment modalities for liver cancer^1^. However, the occurrence of radiation-induced liver disease (RILD) limits the delivery of curative doses of radiation therapy for liver cancer, which is attributed to low tolerance of the liver to radiation^2^. 6.5–17.6% of patients treated with stereotactic body radiotherapy develop RILD, depending on the irradiated liver volume and hepatic functional reserve^3^. As a major complication of radiotherapy for liver cancer, RILD is characterized by hepatocyte death, panlobular congestion, liver fibrosis, and even hepatic dysfunction^4^. RILD hinders the treatment efficiency for liver cancer, which urgently calls for innovative preventive and therapeutic strategies.

The liver is a central immunological organ. As an important trigger of innate and adaptive immunity, toll-like receptor 4 (TLR4) has been recognized as the most critical toll homolog to activate potent immune responses by recognition of endogenous ligands including damage-associated molecular pattern molecules and exogenous ligands, such as lipopolysaccharide (LPS), which is a major component of the outer membrane of Gram-negative bacteria^5^. In the liver, TLR4 is widely expressed in both parenchymal and non-parenchymal cell types and plays an important role in the progress of hepatic injury from a variety of etiologies, including viral hepatitis, metabolic disorder, and ionizing radiation^6^. It was found that irradiation up-regulates the expression of TLR4 in various cell types and promotes the activation of the TLR4 signaling pathway^7^. The TLR4 signal transduction cascade contributes to the secretion of inflammatory factors and the infiltration of inflammatory cells in the microenvironment of the injured liver, resulting in sustained liver inflammation, which promotes the progression of liver injury^8^. A previous study has demonstrated that elevated TLR4 expression in the liver is associated with the development of severe RILD and TLR4 mutant mice have decreased risk of RILD due to a defective TLR4-dependant response^9^. Radiation-induced liver fibrosis is another salient feature of RILD. Hepatic stellate cells (HSCs) are the major fibrogenic cell type in the injured liver, and mediate the progressive accumulation of excessive extracellular matrix proteins, leading to hepatic fibrosis^10^. TLR4 signaling is present in activated HSCs and increases the expression of several pro-inflammatory cytokines, chemokines, and adhesion molecules, linking a series of events between hepatic inflammatory responses and fibrogenesis during liver injury^11^. More importantly, HSCs but not Kupffer cells, have been shown to be the primary targets that drive fibrogenesis in response to TLR4 ligands. Chimeric mice with TLR4 wild-type HSCs and TLR4 mutant Kupffer cells are more sensitive to chemically-induced liver fibrosis compared with TLR4 mutant C3H/HeJ mice and those mice with TLR4 mutant HSCs, but wild-type TLR4 Kupffer cells, indicating the crucial role of TLR4 expression in HSCs^12^. These findings suggest that inhibiting TLR4 expression or blocking its signaling pathway in HSCs may be a novel and effective way to alleviate RILD.

MicroRNAs regulate gene expression after binding to the complementary sequences in the 3′ untranslated regions of the target mRNAs, causing translational repression or cleavage of the target mRNAs^13^. Several miRNAs have been demonstrated to be involved in the regulation of innate immunity^14^. Our previous study showed that microRNA (miR)-146a-5p plays an important role in modulating the LPS/TLR4 pathway involved in the activation of HSCs^15^. In this study, we further explore the functional significance of miR-146a-5p in the regulation of the TLR4 pathway in RILD.

## Results

### Irradiation and LPS stimulation up-regulates the expression of TLR4 pathway genes and miR-146a-5p in LX2 cell

**Fig. 1:**
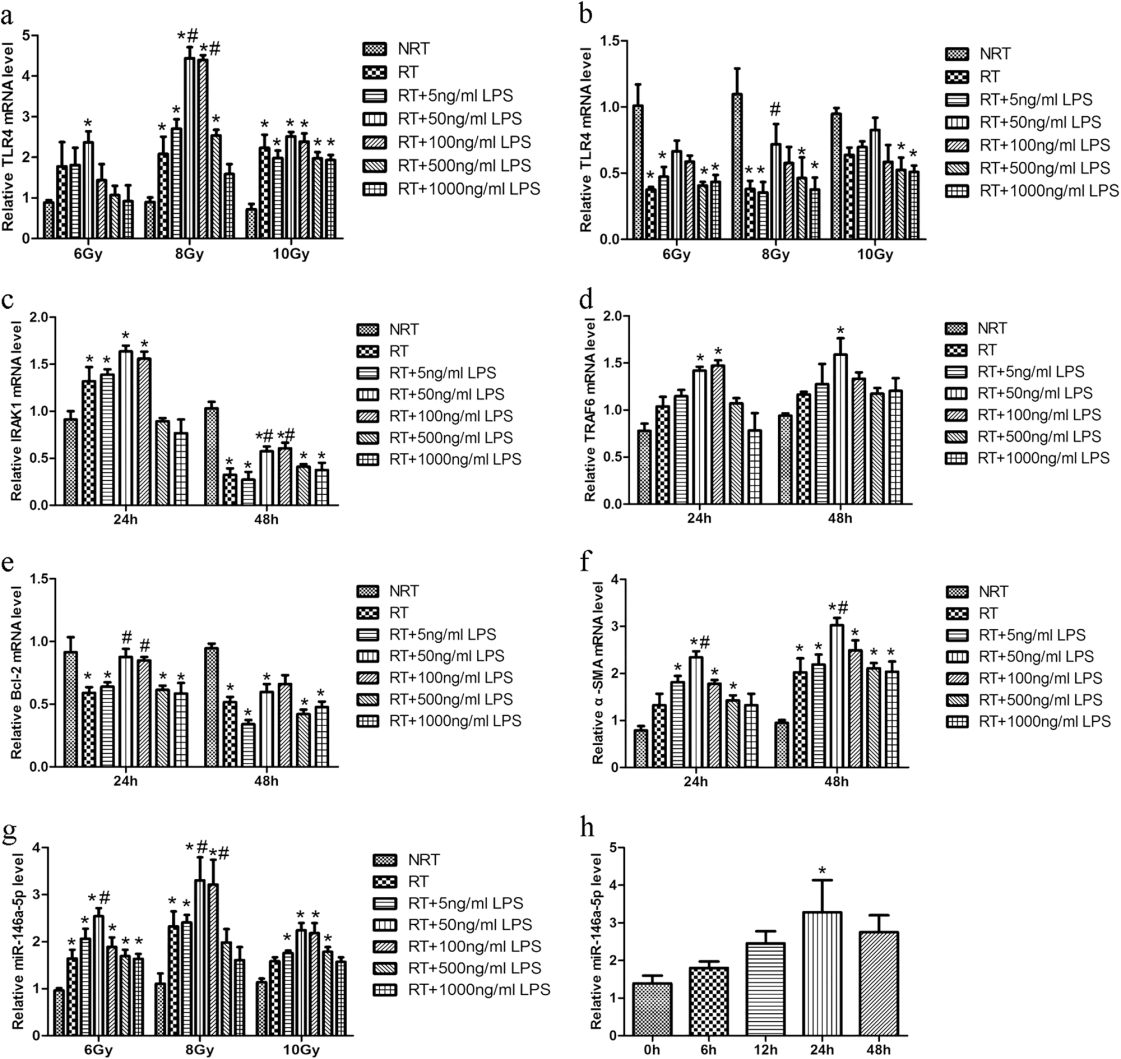
Irradiation and LPS stimulation induces TLR4 and miR-146a-5p expression in LX2 cells. **a** and **b** qRT-PCR analysis showed the expression of TLR4 in LX2 cells at 24 h **a** or 48 h **b** after irradiation and LPS stimulation under various conditions, as indicated. **c**–**f** LX2 cells were treated with 8 Gy X-ray irradiation and 50 ng/ml LPS for 24 or 48 h and then qRT-PCR was performed to detect the expression levels of IRAK1 **c**, TRAF6 **d**, Bcl-2 **e** and α-SMA **f.** **g** miR-146a-5p expression was examined by qRT-PCR at 24 h after irradiation and LPS stimulation under various conditions, as indicated. **h** miR-146a-5p expression was assessed by qRT-PCR after treatment with 8 Gy X ray irradiation and 50 ng/ml LPS for the indicated time periods. All data are presented as the mean ± S.E.M. of three independent experiments. *P < 0.05 vs. NRT group; ^#^*P* < 0.05 vs. RT group. *NRT* non-irradiation treatment, *RT* irradiation treatment

To explore the effect of irradiation and LPS on the expression of TLR4 and miR-146a-5p, LX2 cells were treated with different doses of X-ray irradiation (6–10 Gy) and various amounts of LPS (0–1000 ng/ml) for 24 and 48 h. Compared with non-irradiated LX2 cells, the TLR4 expression levels were significantly up-regulated in LX2 cells irradiated with 8 or 10 Gy for 24 h. Additionally, 8 Gy irradiation combined with low-dose LPS (50–100 ng/ml) stimulation for 24 h further increased the expression of TLR4 in LX2 cells, whereas these effects were not observed in case of 10 Gy irradiation (Fig. 1a). However, the expression levels of TLR4 were down-regulated in LX2 cells at 48 h after irradiation with or without LPS treatment, as compared with non-irradiated cells (Fig. 1b). Similarly, as the downstream gene of TLR4, interleukin (IL)-1 receptor-associated kinase 1 (IRAK1) and tumor necrosis factor (TNF) receptor associated factor-6 (TRAF6) were significantly up-regulated in LX2 cells subjected to 8 Gy irradiation and 50 ng/ml LPS stimulation for 24 h (Figs. 1c and d). Compared with LX2 cells treated with irradiation alone, those treated additionally with LPS showed up-regulated expression of Bcl-2 (Fig. 1e). Moreover, α-smooth muscle actin (α-SMA), the activation marker of LX2 cells, was markedly increased at 24 h after combined treatment with irradiation and LPS and further increased at 48 h (Fig. 1f). In line with the change in TLR4 expression, under conditions of 8 Gy irradiation and 50 ng/mL LPS treatment for 24 h, miR-146a-5p level was also significantly up-regulated and reached a peak compared with control (Figs. 1g and h). These results indicate a correlation between miR-146a-5p, TLR4 downstream gene expression and cell activation in irradiated and LPS-stimulated LX2 cells.

### Overexpression of miR-146a-5p suppresses cell proliferation, pro-inflammatory cytokine production, and cell activation in irradiated and LPS-stimulated LX2 cells

**Fig. 2:**
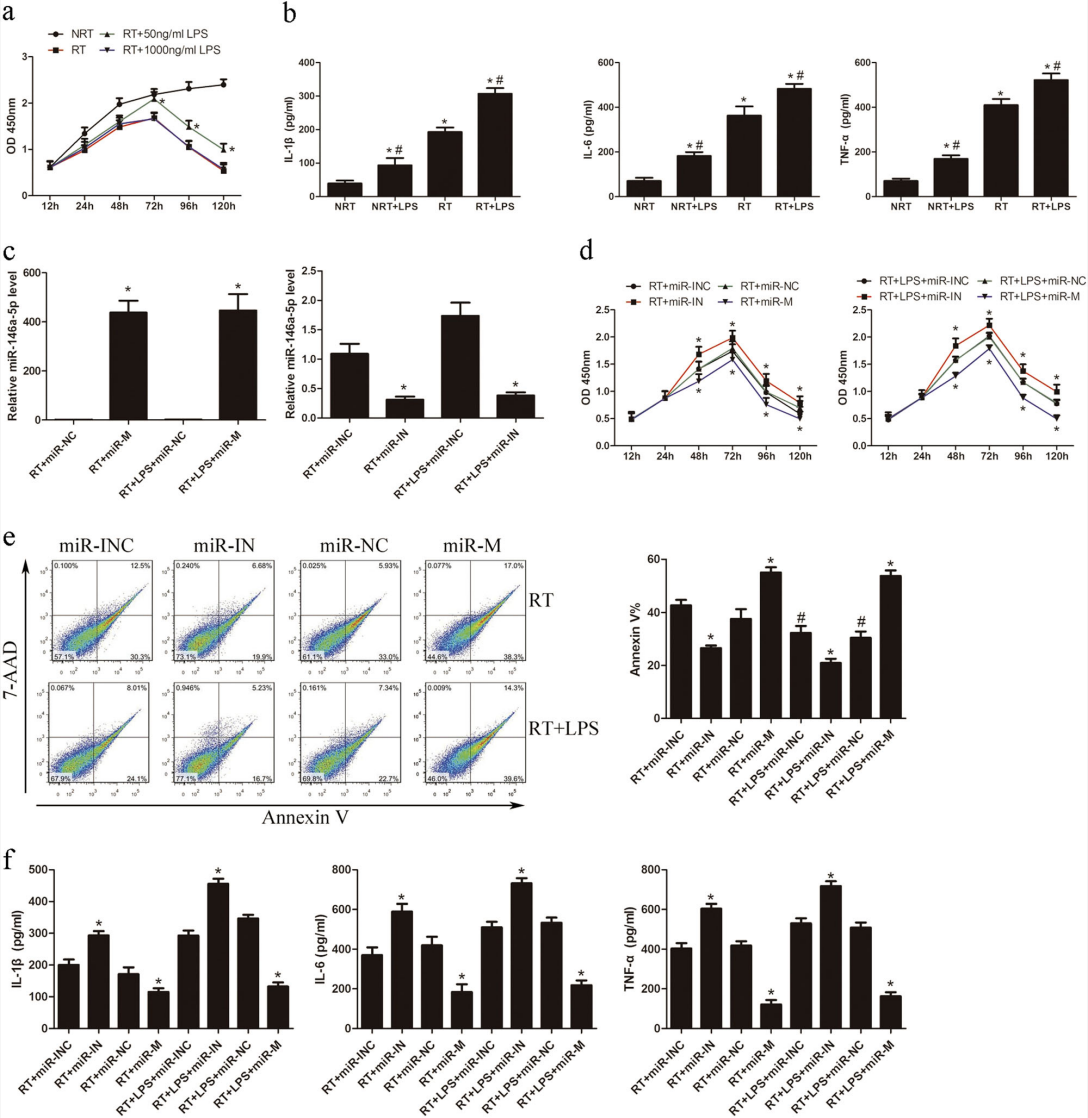
Overexpression of miR-146a-5p inhibits cell proliferation, pro-inflammatory cytokines secretion, and cell activation in irradiated and LPS-stimulated LX2 cells. LX2 cells were subjected to 8 Gy X-ray irradiation with or without 50 ng/ml LPS treatment. **a** CCK8 assay showed the proliferation of LX2 cells in the presence or absence of irradiation and LPS treatment. *P < 0.05 vs. RT group. **b** Secretion levels of IL-1β, IL-6, and TNF-α in LX2 cells with or without irradiation and LPS treatment. *P < 0.05 vs. NRT group; ^#^*P* < 0.05 vs. RT group. **c** Expression of miR-146a-5p after transfection of LX2 cells with miR-146a-5p mimics or inhibitors compared to their corresponding negative control. *P < 0.05 vs. corresponding negative control. **d** CCK8 assay showed the proliferation of LX2 cells with altered expression of miR-146a-5p. *P < 0.05 vs. corresponding negative control. **e** Apoptosis analysis of LX2 cells transfected with miR-146a-5p mimics or inhibitors. *P < 0.05 vs. corresponding negative control; ^#^*P* < 0.05 vs. corresponding RT group. **f** Secretion levels of IL-1β, IL-6, and TNF-α in LX2 cells with altered expression of miR-146a-5p. *P < 0.05 vs. corresponding negative control. All data are presented as the mean ± S.E.M. of three independent experiments. *miR-INC* miRNA inhibitors negative control, *miR-IN* miRNA inhibitors, *miR-NC* miRNA negative control, *miR-M* miRNA mimics

**Fig. 3:**
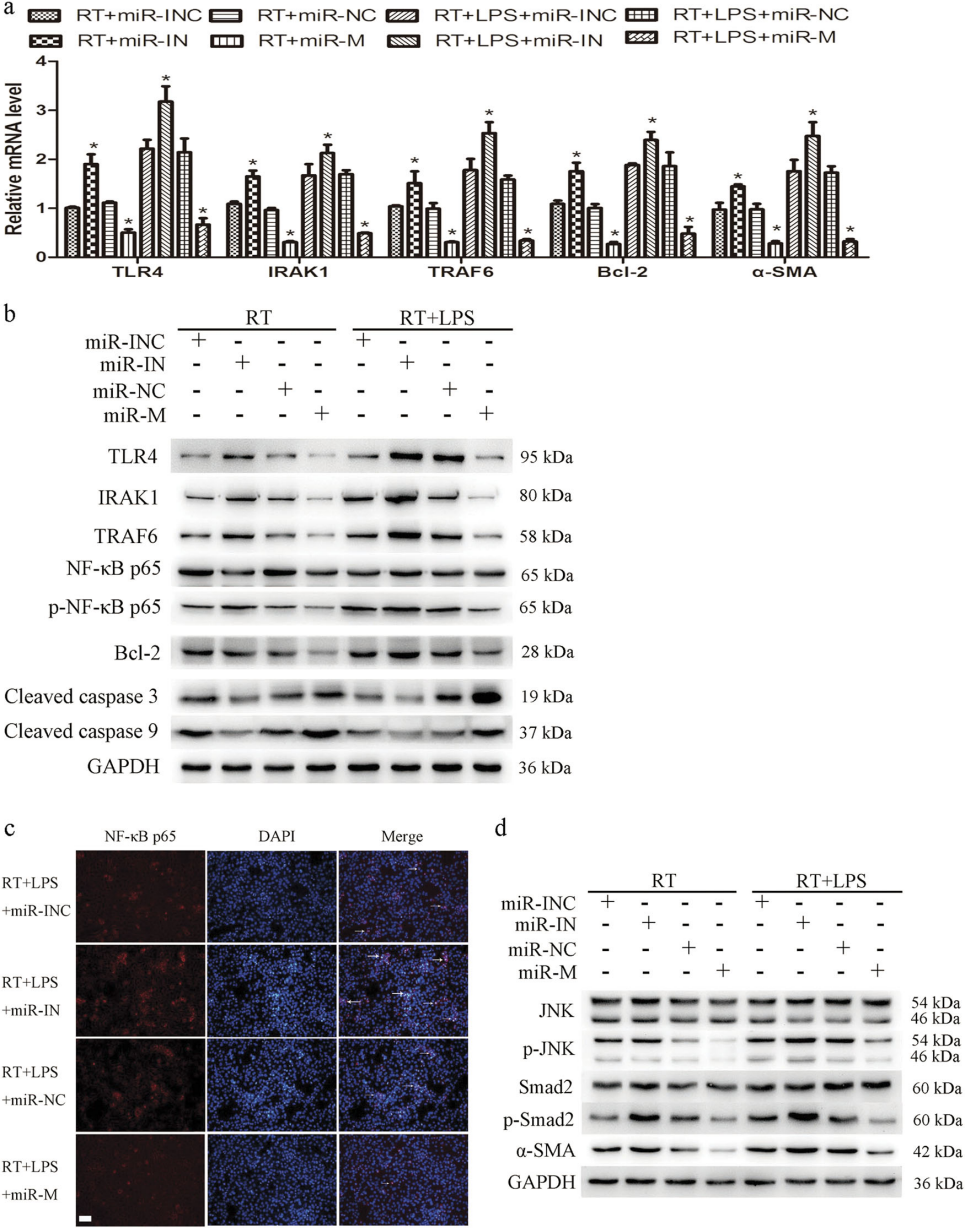
Overexpression of miR-146a-5p inhibited the activation of TLR4 signaling in LX2 cells after irradiation and LPS stimulation. LX2 cells were transfected with miR-146a-5p mimics or inhibitors and then subjected to 8 Gy X-ray irradiation with or without 50 ng/ml LPS treatment. **a** qRT-PCR analysis showed the expression levels of TLR4, its downstream genes, and α-SMA in LX2 cells. Data are presented as the mean ± S.E.M. of three independent experiments. *P < 0.05 vs. corresponding negative control. **b** Representative western blots showed the expression of TLR4, IRAK1, and TRAF6, phosphorylation of NF-κB p65, and the expression of apoptosis-related proteins in LX2 cells. **c** Immunofluorescence staining showed NF-κB p65 nuclear translation (indicated by arrows) in LX2 cells. Scale bar: 100 μm. **d** Representative western blots showed the phosphorylation of JNK and Smad2 and the expression of α-SMA in LX2 cells. *miR-INC* miRNA inhibitors negative control, *miR-IN* miRNA inhibitors, *miR-NC* miRNA negative control, *miR-M* miRNA mimics, *TLR4* toll-like receptor 4, *IRAK1* IL-1 receptor associated kinase 1, *TRAF6* tumor necrosis factor receptor associated factor 6, *NF-κB* nuclear factor-kappa B, *α-SMA* α-smooth muscle actin

Since a low dose of LPS significantly enhanced the expression of TLR4 signaling genes in irradiated LX2 cells, we further investigated the effect of irradiation and low-dose LPS treatment on the biological function of LX2 cells. As shown in Fig. 2a, low-dose LPS treatment promoted the proliferation of irradiated LX2 cells compared with high-dose LPS treatment. Additionally, while irradiation for 24 h significantly increased the production of pro-inflammatory cytokines, including IL-1β, IL-6, and TNF-α, irradiation combined with low-dose LPS stimulation for 24 h further enhanced this effect, as compared with non-irradiated cells (Fig. 2b). Considering miR-146a-5p is a key regulator of innate immune responses, we determined the regulatory effects of miR-146a-5p on LX2 cells. Cells were transiently transfected with miR-146a-5p mimics or inhibitors to elevate or inhibit the expression of miR-146a-5p (Fig. 2c), and then subjected to 8 Gy irradiation with or without 50 ng/ml LPS treatment for the indicated time. Results showed that ectopic expression of miR-146a-5p significantly suppressed cell proliferation and induced cell apoptosis (Figs. 2d and e). Overexpression of miR-146a-5p significantly inhibited pro-inflammatory cytokines production in LX2 cells (Fig. 2f). Moreover, pretreatment with miR-146a-5p mimics also significantly suppressed α-SMA mRNA expression in LX2 cells (Fig. 3a). However, transfection with miR-146a-5p inhibitors showed contrary changes in LX2 cells. These results indicate that miR-146a-5p ectopic expression can significantly decrease proliferation ability, pro-inflammatory cytokines production, and cell activation in LX2 cells.

### MiR-146a-5p negatively regulates irradiation-induced and LPS-induced TLR4 signaling pathway activation

Having determined the regulatory effect of miR-146a-5p on LX2 cells, we next explored the involvement of miR-146a-5p in the TLR4 pathway. After transfection with miR-146a-5p inhibitors or mimics, LX2 cells were subjected to 8 Gy irradiation with or without 50 ng/ml LPS treatment for 24 h. Compared with control, the mRNA levels of TLR4, IRAK1, and TRAF6 were significantly decreased after transfection with miR-146a-5p mimics (Fig. 3a). Western blots also showed that miR-146a-5p mimics significantly reduced the expression of TLR4, IRAK1, and TRAF6, as well as the phosphorylation of nuclear factor-kappa B (NF-κB) in LX2 cells (Fig. 3b). Immunofluorescence staining revealed that nuclear translocation of NF-κB induced by irradiation and LPS stimulation in LX2 cells was inhibited by transfection with miR-146a-5p mimics (Fig. 3c). Considering that activation the p65 subunit of NF-κB is important in regulating anti-apoptosis gene expression, we confirmed that reduced phosphorylation of NF-κB p65 via overexpression of miR-146a-5p markedly inhibits the expression of Bcl-2 and increases the cleaved caspase 3 and cleaved caspase 9 levels in LX2 cells (Fig. 3b). Furthermore, compared to control, pretreatment with miR-146a-5p mimics significantly attenuated irradiation-induced and LPS-induced α-SMA production via decrease of phosphor(p)-JNK and p-Smad2 expression in LX2 cells (Fig. 3d). Conversely, inhibition of miR-146a-5p exerted opposite effects. These results collectively suggest that miR-146-5p negatively regulates irradiation and LPS induced TLR4 pathway activation.

### The regulatory effects of miR-146a-5p on LX2 cells are mediated through IRAK1 and TRAF6

**Fig. 4:**
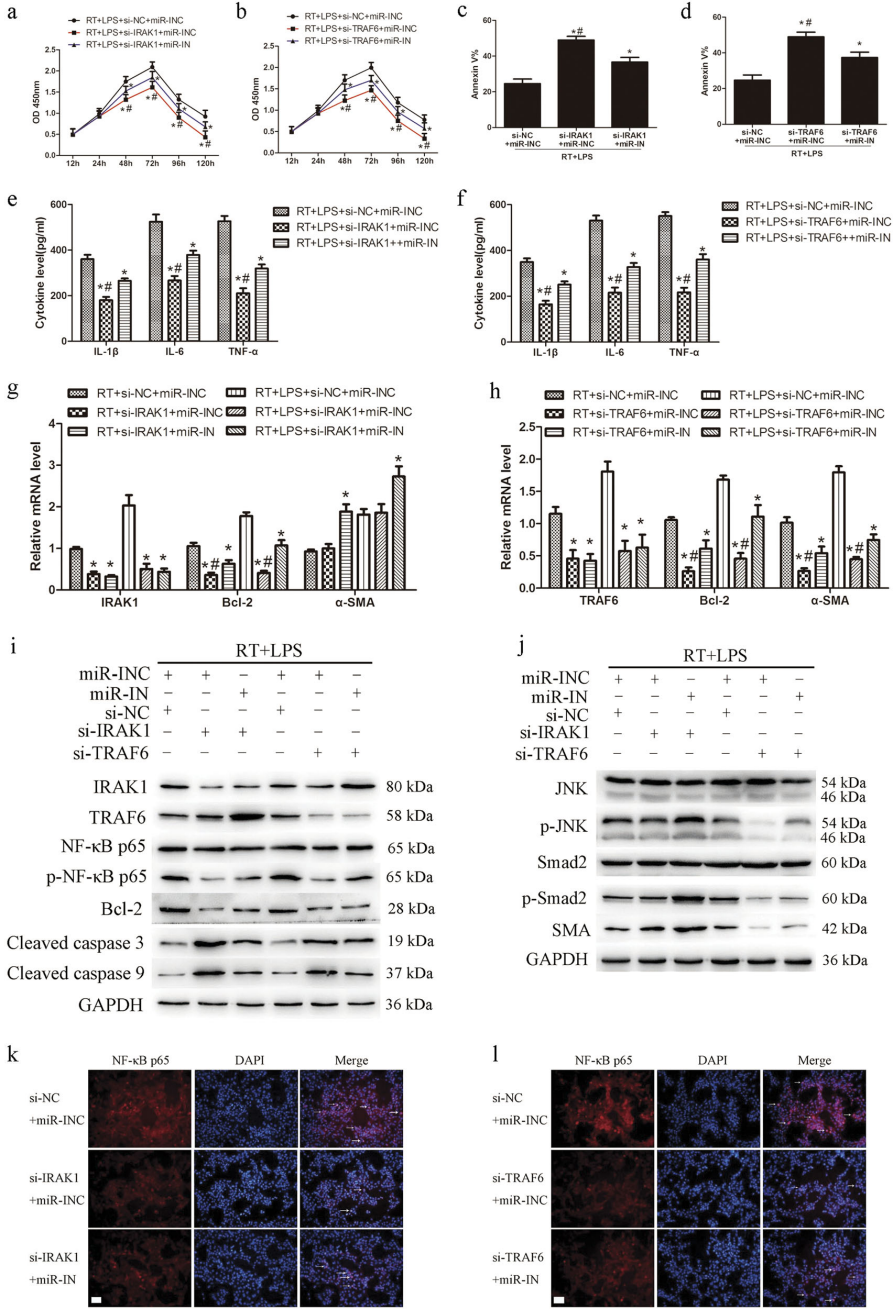
Knockdown of IRAK1 or TRAF6 suppressed the production of pro-inflammatory cytokines and cell activation in irradiated and LPS-stimulated LX2 cells. LX2 cells were treated with siRNA against IRAK1 or TRAF6 together with miR-146a-5p inhibitors and then subjected to 8 Gy X-ray irradiation and 50 ng/ml LPS treatment. **a** and **b** CCK8 assay showed the proliferation of LX2 cells transfected with si-IRAK1 **a** or si-TRAF6 **b**. **c** and **d** Apoptosis analysis of LX2 cells transfected with si-IRAK1 **c** or si-TRAF6 (**d**). **e** and **f** Secretion levels of IL-1β, IL-6, and TNF-α in LX2 cells transfected with si-IRAK1 **e** or si-TRAF6 **f**. **g** and **h** qRT-PCR analysis showed the expression of IRAK1, TRAF6, Bcl-2, or α-SMA in LX2 cells transfected with si-IRAK1 **g** or si-TRAF6 **h**. **i** Representative western blots showed the expression of IRAK1 and TRAF6, phosphorylation of NF-κB p65, and the expression of apoptosis-related proteins in LX2 cells. **j** Representative western blots showed the phosphorylation levels of JNK and Smad2 and the expression of α-SMA in LX2 cells. **k** and **l** Immunofluorescence staining showed NF-κB p65 nuclear translation (indicated by arrows) in LX2 cells transfected with si-IRAK1 **k** or si-TRAF6 **l**. Scale bar: 100 μm. All data are presented as the mean ± S.E.M. of three independent experiments. *P < 0.05 vs. corresponding negative control; ^#^*P* < 0.05 vs. corresponding miR-IN group. *si-NC* siRNA negative control for IRAK1 or TRAAF6, *si-IRAK1* siRNA specifically against IRAK1, *si-TRAF6* siRNA specifically against TRAF6, *miR-INC* miRNA inhibitors negative control, *miR-IN* miRNA inhibitors

To decipher whether IRAK1 and TRAF6 mediate the regulatory effects of miR-146a-5p in LX2 cells, LX2 cells were co-transfected with IRAK1 or TRAF6 small interfering RNA (siRNA) and miR-146a-5p inhibitors. The cells were then subjected to 8 Gy irradiation and 50 ng/ml LPS treatment for the indicated time. Notably, respective knockdown of IRAK1 and TRAF6 markedly inhibited the proliferation of LX2 cells (Figs. 4a and b). IRAK1 or TRAF6 siRNA transfection also promoted LX2 cell apoptosis at 72 h after treatment (Figs. 4c and d). Silencing of IRAK1 or TRAF6 decreased the pro-inflammatory cytokines production in LX2 cells after treatment for 24 h (Figs. 4e and f). Mechanism study revealed that knockdown of IRAK1 or TRAF6 inhibited NF-κB p65 phosphorylation, reduced Bcl-2 and increased cleaved caspase 3 and cleaved caspase 9 expression in LX2 cells (Figs. 4g-i). Knockdown of IRAK1 or TRAF6 also inhibited the nuclear translocation of NF-κB p65 in LX2 cells (Figs. 4k and l). Furthermore, siRNA silencing of TRAF6 rather than IRAK1 significantly inhibited irradiation-induced and LPS-induced JNK and Smad2 phosphorylation, and reduced α-SMA expression (Figs. 4g, h, and j). However, the above mentioned regulatory effects of IRAK1 or TRAF6 siRNA on LX2 cells were partly attenuated by co-transfection with miR-146a-5p inhibitors. These results collectively indicate that IRAK1 and TRAF6 are required for the regulatory effect of miR-146a-5p on LX2 cells.

### Culture medium from irradiated and LPS-stimulated LX2 cells with miR-146a-5p overexpression could attenuate irradiation induced hepatocyte apoptosis

**Fig. 5:**
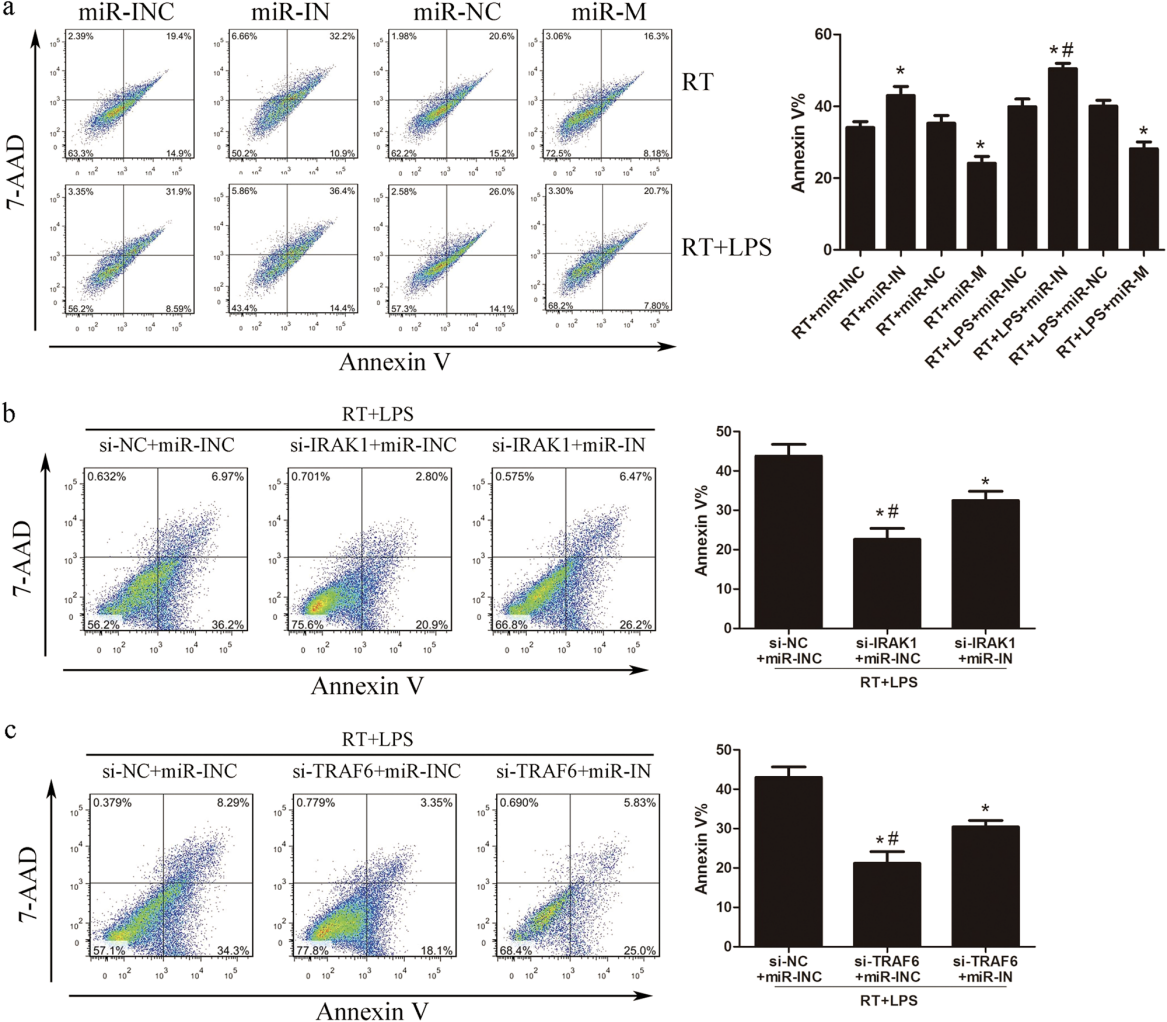
Culture supernatants from irradiated and LPS-stimulated LX2 cells transfected with miR-146a-5p mimics could alleviate irradiation-induced hepatocyte apoptosis. **a** Apoptosis analysis of LO2 cells at 72 h after irradiation and co-culture with supernatants from irradiated and LPS-stimulated LX2 cells transfected with miR-146a-5p mimics or inhibitors. *P < 0.05 vs. corresponding negative control; ^#^*P* < 0.05 vs. RT + miR-IN group. **b** and **c** Apoptosis analysis of LO2 cells at 72 h after irradiation and co-culture with supernatants from irradiated and LPS-stimulated LX2 cells transfected with siRNA against IRAK1 **b** or TRAF6 **c** together with miR-146a-5p inhibitors. *P < 0.05 vs. corresponding negative control; ^#^*P* < 0.05 vs. si-IRAK1 or si-TRAF6 + miR-IN group. Data are presented as the mean ± S.E.M. from three independent experiments. *si-NC* siRNA negative control for IRAK1 or TRAAF6, *si-IRAK1* siRNA specifically against IRAK1, *si-TRAF6* siRNA specifically against TRAF6, *miR-INC* miRNA inhibitors negative control, *miR-IN* miRNA inhibitors, *miR-NC* miRNA negative control, *miR-M* miRNA mimics

Given that irradiation and LPS treatment could induce pro-inflammatory cytokines production in LX2 cells, we further investigated hepatocyte survival after irradiation and incubation with the culture medium from irradiated and LPS stimulated LX2 cells for 72 h. Compared with the morphology of LO2 cells before treatment, the morphological characteristics of apoptosis, including nuclear condensation and cell shrinkage, were most evident in irradiated LO2 cells cultured with the supernatants of LX2 cells transfected with miR-146a-5p inhibitors. LO2 cells cultured with the supernatants of cells transfected with miR-146a-5p mimics showed lesser evident morphological characteristics of apoptosis (Supplementary Fig. 1a). In addition, the supernatant levels of aspartate transaminase (AST) and alanine aminotransferase (ALT) were significantly higher in those irradiated LO2 cells cultured with the supernatants from miR-146a-5p inhibitors transfected LX2 cells, but were significantly lower in irradiated LO2 cells cultured with the supernatants of LX2 cells transfected with miR-146a-5p mimics, as compared with control cells (Supplementary Fig. 1b). Flow cytometry analysis showed that, compared with negative control group, the culture medium from LX2 cells with miR-146a-5p overexpression alleviated the apoptosis of irradiated LO2 cells, while that from miR-146a-5p inhibitors-transfected LX2 cells further enhanced the apoptosis of LO2 cells (Fig. 5a). Moreover, the culture medium from LX2 cells transfected with IRAK1 or TRAF6 siRNA also achieved the same attenuation effect on LO2 apoptosis after irradiation. Furthermore, the decreased LO2 cell apoptosis was mitigated by co-transfection with miR-146a-5p inhibitors (Figs. 5b and c). These results suggest that miR-146a-5p plays an important role in modulating HSC microenvironment to influence irradiation-induced hepatocyte apoptosis.

### miR-146a-5p alleviates irradiation-induced liver disease in mice through inhibition of the TLR4 signaling pathway

**Fig. 6:**
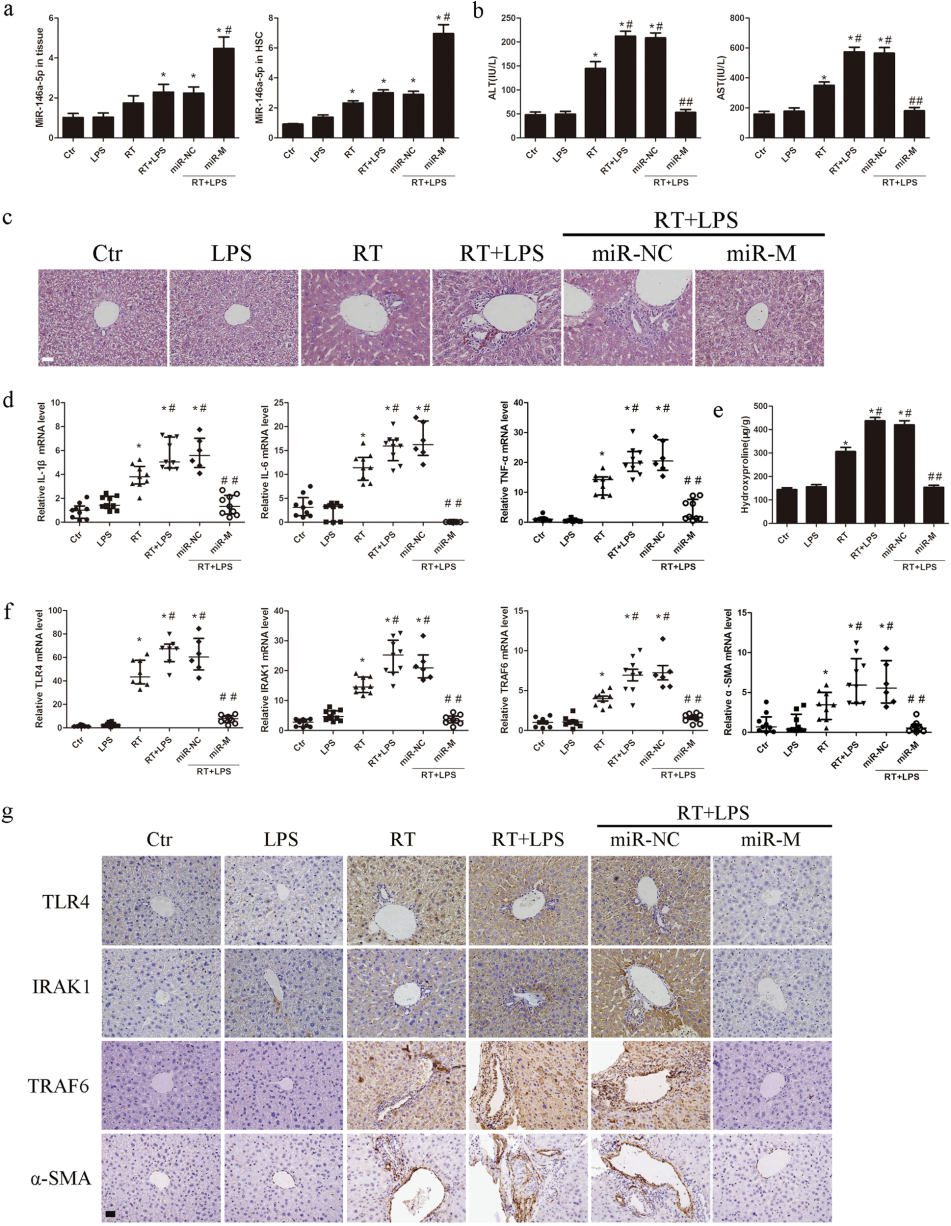
miR-146a-5p attenuates RILD in mice. **a** qRT-PCR analysis showed the expression levels of miR-146a-5p in the liver tissue and primary HSCs of mice in each group. Data are presented as the mean ± standard deviation (SD) (*n* = 3 mice). *P < 0.05 vs. Ctr group; ^#^*P* < 0.05 vs. miR-NC group. **b** Serum levels of ALT and AST in mice of each group. Data are presented as the mean ± SD (*n* = 6 mice). *P < 0.05 vs. Ctr group; ^#^*P* < 0.05 vs. RT group; ^##^*P* < 0.05 vs. miR-NC group. **c** Representative HE staining of the liver tissue of mice subjected different treatments, as indicated. Scale bar: 100 μm. **d** qRT-PCR analysis showed the expression levels of pro-inflammatory cytokines in the liver tissue of mice in each group. Data are presented as the median with interquartile range (*n* = 6-9 mice). *P < 0.05 vs. Ctr group; ^#^*P* < 0.05 vs. RT group; ^##^*P* < 0.05 vs. miR-NC group. **e** Hepatic hydroxyproline content in mice of each group. Data are presented as the mean ± SD (*n* = 6 mice). *P < 0.05 vs. Ctr group; ^#^*P* < 0.05 vs. RT group; ^##^*P* < 0.05 vs. miR-NC group. **f** qRT-PCR analysis showed the expression levels of TLR4, IRAK1, TRAF6, and α-SMA in the liver tissue of mice in each group. Data are presented as the median with interquartile range (*n* = 6–9 mice). *P < 0.05 vs. Ctr group; ^#^*P* < 0.05 vs. RT group; ^##^*P* < 0.05 vs. miR-NC group. **g** Representative immunohistochemical staining showed the expression of TLR4, IRAK1, TRAF6, and α-SMA in the liver tissue of mice in each group. Scale bar: 100 μm. *Ctr* blank control, *miR-NC* miRNA negative control, *miR-M* miRNA mimics

**Fig. 7:**
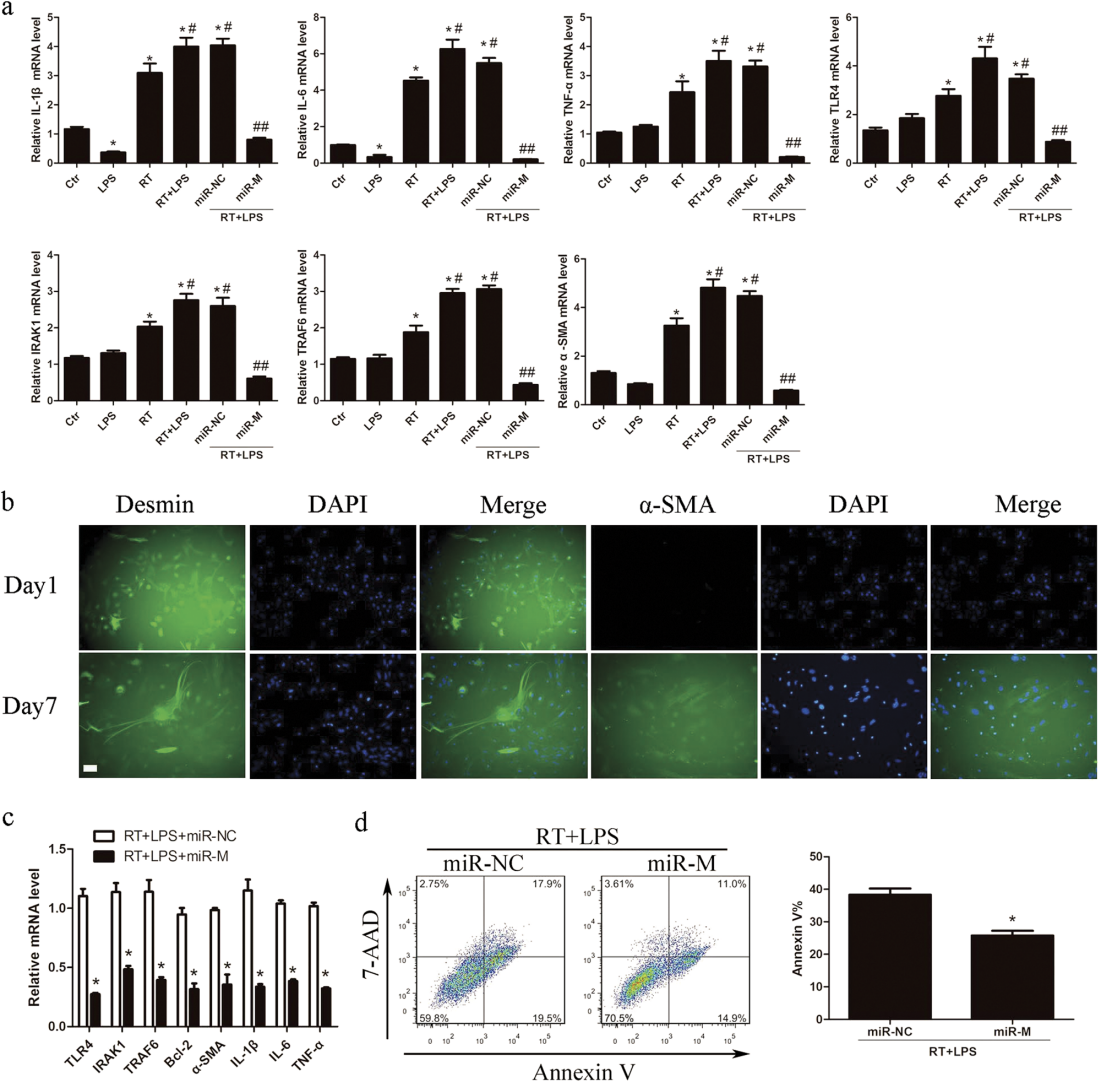
miR-146a-5p negatively regulates TLR4 signaling in primary HSCs. **a** qRT-PCR analysis showed the expression levels of pro-inflammatory cytokines, TLR4, IRAK1, TRAF6, and α-SMA in primary HSCs of mice in each group. Data are presented as the mean ± SD (*n* = 3 mice). *P < 0.05 vs. Ctr group; ^#^*P* < 0.05 vs. RT group; ^##^*P* < 0.05 vs. miR-NC group. **b** Representative immunofluorescence staining of desmin and α-SMA for cultured primary HSCs at the first day and 7 days after isolation. Scale bar: 100 μm. **c** Quiescent HSCs (24 h after isolation) were transfected with miR-146a-5p mimics or negative control and then subjected to 8 Gy X-ray irradiation and 50 ng/ml LPS treatment for 24 h. The expression levels of TLR4, IRAK1, TRAF6, pro-inflammatory cytokines, and α-SMA were detected by qRT-PCR. Data are presented as the mean ± S.E.M. of three independent experiments. *P < 0.05 vs. miR-NC group. **d** Apoptosis analysis of primary hepatocytes at 72 h after 8 Gy X-ray irradiation and co-culture with the supernatants from irradiated and LPS-stimulated primary HSCs transfected with miR-146a-5p mimics or negative control. Data are presented as the mean ± S.E.M. of three independent experiments. *P < 0.05 vs. miR-NC group. *miR-NC* miRNA negative control, *miR-M* miRNA mimics

miR-146a-5p mimics or negative control was administered to C3H/HeN mice with a single dose of 30 Gy X-ray irradiation in the liver and LPS daily administration. Compared with negative control, miR-146a-5p was up-regulated in the mice liver tissues and primary HSCs in miR-146-5p treatment group (Fig. 6a). Compared with untreated mice, daily LPS administration or irradiation or combination treatment could increase the serum levels of LPS, which were not affected by miR-146a-5p treatment (Supplementary Fig. 2a). The serum levels of AST and ALT were increased markedly in irradiated mice, particularly in the irradiation and daily LPS administration group, but were significantly attenuated by miR-146a-5p treatment (Fig. 6b). According to hematoxylin-eosin-stained liver sections, intralobular spotty necrosis and/or inflammatory cell infiltration around the vasculature in mice with irradiation and daily LPS administration were found to be more serious than that in mice with irradiation alone. These histopathological changes of RILD were significantly improved in miR-146a-5p treatment group (Fig. 6c). Irradiation with or without daily LPS administration also induced the production of pro-inflammatory cytokines in the liver tissues and primary HSCs, which was suppressed by miR-146a-5p treatment (Figs. 6d and 7a). In addition, the hepatic hydroxyproline content was significantly increased in irradiated mice, and further elevated in irradiation and daily LPS administration group, but was significantly attenuated in miR-146a-5p treatment group (Fig. 6e). Further experiments showed that irradiation and daily LPS administration induced TLR4, IRAK1, TRAF6, and α-SMA expression in the liver tissues and primary HSCs, but were notably suppressed in miR-146a-5p treatment group (Figs. 6f and g, Fig. 7a). Reduced expression of pro-apoptotic and pro-fibrotic proteins in the liver tissues were also observed in miR-146a-5p treatment group (Supplementary Fig. 2b–e).

**Fig. 8:**
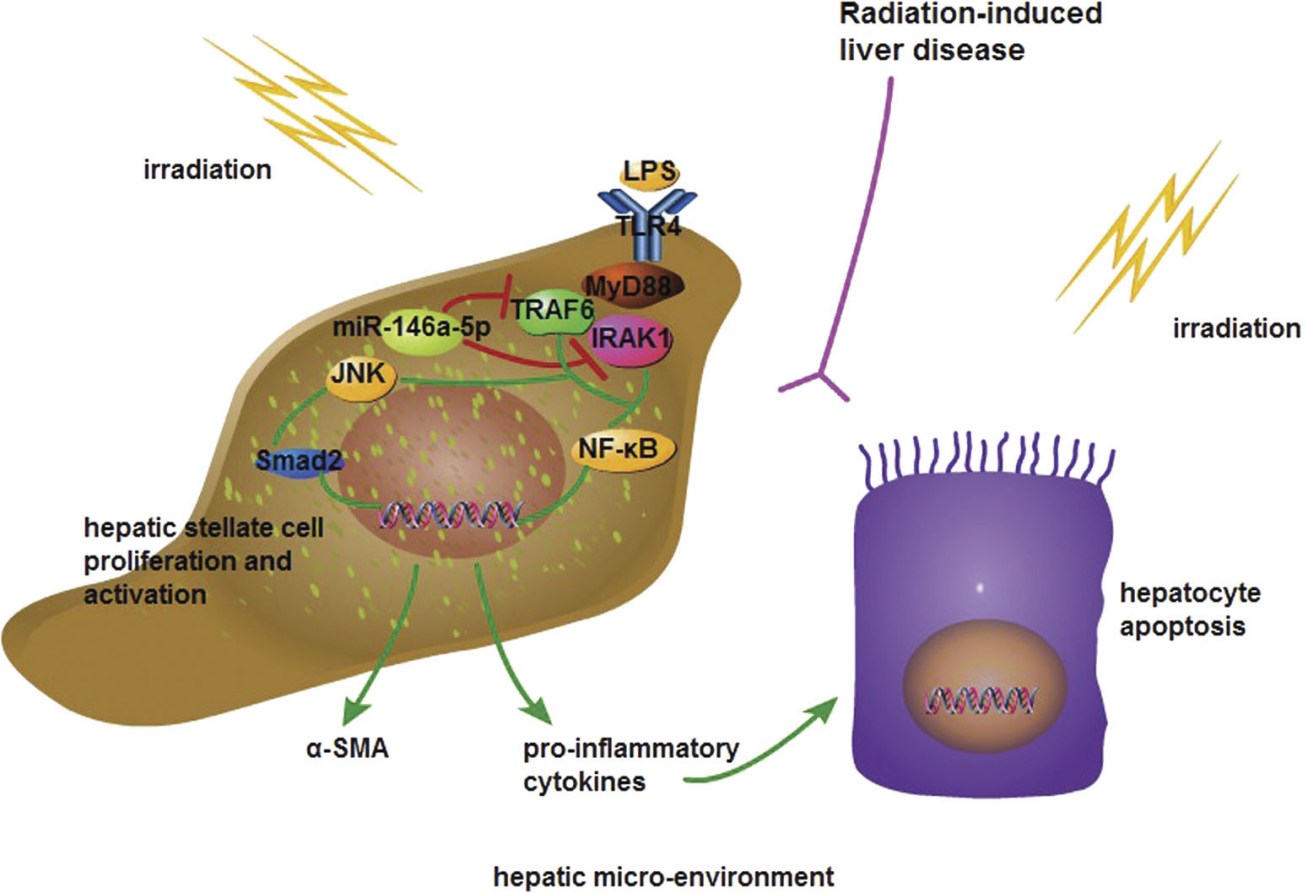
Proposed model of miR-146a-5p involved in attenuating the progress of RILD via downregulation of TLR4 signaling. MiR-146a-5p downregulates TLR4 pathway to suppress irradiation-induced and LPS-induced pro-inflammatory cytokines production and HSC activation, as well as alleviate radiation-induced hepatocyte injury

Furthermore, the primary quiescent HSCs and hepatocytes were isolated from mice for investigation. The cultured primary HSCs were activated on day 7 as detected by immunofluorescence staining (Fig. 7b), and thus the primary quiescent HSCs (24 h after isolation) were used for following experiments. By pretreatment with miR-146a-5p mimics, the expression levels of TLR4, IRAK1, TRAF6, α-SMA, and pro-inflammatory cytokines were significantly down-regulated in irradiated and LPS-stimulated primary HSCs (Fig. 7c). When co-cultured with the culture medium from the irradiated and LPS-stimulated primary HSCs transfected with miR-146a-5p mimics, the apoptosis rates of primary hepatocytes at 72 h after irradiation were decreased compared with negative control group (Fig. 7d). Interestingly, compared with negative control group, miR-146a-5p treatment group did not show an increase in miR-146a-5p level in primary hepatocytes from mice with irradiation and daily LPS administration (Supplementary Fig. 3a). Moreover, transfection with miR-146a-5p mimics had no effect on the apoptosis of primary hepatocytes after irradiation with or without LPS stimulation (Supplementary Fig. 3b). These results indicate that miR-146a-5p alleviates irradiation-induced liver disease in mice partly through inhibition of the TLR4 signaling pathway in HSCs (Fig. 8).

## Discussion

Previous studies have demonstrated an important role of TLR4 signaling in liver injury and fibrogenesis and an association between high TLR4 expression and RILD risk^6,9^. In this study, we explored the effect of irradiation and LPS on the induction of TLR4 expression in LX2 cells. Hepatic non-parenchymal cells, including HSCs, are not sensitive to radiation in vitro and would not be expected to develop notable immune response and cell damage if the radiation dose is less than 8-10 Gy^16^. Thus, 6 Gy irradiation could not efficiently induce the expression of TLR4 in LX2 cells. We found that 8 Gy irradiation combined with a low dose of LPS (50-100 ng/ml) significantly elevated the expression of TLR4 in LX2 cells, but these inductive effects were modest in 10 Gy irradiation combined with LPS treatment groups, which might be attributed to the high dose of irradiation causing more cell damage and death, resulting in the downregulation of TLR4 expression. Moreover, the inductive effects of irradiation combined with a high dose of LPS (500–1000 ng/ml) on TLR4 expression in LX2 cells were not as significant as that combined with a low dose of LPS. Similar results of TLR4 expression have been observed in macrophages treated with irradiation combined with low or high dose of LPS, respectively^7^. Downregulation of TLR4 expression under conditions of irradiation and high-dose LPS treatment might be ascribed to LPS tolerance. Large amounts of LPS are usually well tolerated within the healthy intestine^17^. In mouse peritoneal macrophages, it was demonstrated that downregulation of TLR4 expression is responsible for LPS tolerance^18^.

miRNAs have emerged as key regulators of inflammatory responses and fibrogenesis^14,19^. In this study, we observed that miR-146a-5p expression was changed in line with TLR4 after irradiation and LPS stimulation, indicating that a potential regulation mechanism between TLR4 and miR-146a-5p might exist. Indeed, the MyD88-dependent pathway mediates LPS/TLR4 signaling via recruitment of IRAK1 and TRAF6 to promote the activation of NF-κB and controls the expression of pro-inflammatory cytokines. By means of promoter analysis, it has been shown that miR-146a-5p expression induced by activation of TLR4 signaling occurs in an NF-κB dependent manner in several kinds of cells^20,21^. This might be a potential explanation for the up-regulation of miR-146a-5p in response to TLR4 in this study. More importantly, IRAK1 and TRAF6 have been confirmed to be the unequivocal targets of miR-146a-5p in several studies^22,23^. Our study corroborates other reports that ectopic expression of miR-146-5p inhibits in vitro pro-inflammatory cytokines production by decreasing the expression levels of IRAK1 and TRAF6 and by inactivation of NF-κB^24,25^. This indicates that induction of miR-146a-5p expression by irradiation and LPS stimulation appears to elicit a negative feedback regulation, which modulates the TLR4 pathway. Furthermore, knockdown of IRAK1 and TRAF6 produced a similar biological outcome, with regard to miR-146a-5p, in repressing the production of pro-inflammatory cytokines. This effect was partly rescued by inhibition of miR-146a-5p, suggesting that IRAK1 and TRAF6 are the main functional targets of miR-146a-5p in the regulation of irradiation-induced and LPS-induced pro-inflammatory effects.

It was reported that LPS stimulates the synthesis of TNF-α and IL-6 in both quiescent and activated HSCs, and the culture medium from LPS-challenged HSCs can induce apoptosis of cultured hepatocytes^26–28^. In this study, the culture medium from irradiated-stimulated and endotoxin-stimulated HSCs with miR-146a-5p overexpression significantly alleviated apoptosis of irradiated hepatocytes, which might be partly ascribed to the reduced production of pro-inflammatory cytokines by HSCs. Likewise, irradiation-induced and LPS-induced pro-inflammatory cytokines production and inflammatory cells infiltration were markedly attenuated in mice treated with miR-146a-5p, indicating miR-146a-5p treatment decreased the release of pro-inflammatory cytokines into the hepatic microenvironment. Although Kupffer cells are considered to be important targets of TLR ligands in the liver, emerging evidence reveals that HSCs are essential for mediating hepatic inflammatory responses during liver injury. After intraperitoneally injecting LPS in Kupffer cell–depleted mice, nuclear translocation of NF-κB p65 is still detected in a large number of HSCs, whereas this phenomenon is not observed in hepatocytes and desmin-negative non-parenchymal cells, indicating that HSCs are targets of LPS in vivo^12^. Analogously, after depletion of HSCs in mice, acute hepatocyte injury and TNF-α levels in the liver are significantly reduced in LPS-induced liver injury, suggesting that HSCs contribute to endotoxin-induced hepatocyte death and play an important role in mediating TNF-α production during liver injury in vivo^29^. We also confirmed that the expression of IL-1β, IL-6, and TNF-α is elevated in HSCs isolated from mice treated with irradiation combined with LPS. Taken together, miR-146a-5p alleviates irradiation-induced and LPS-induced hepatocyte injury in vitro and in vivo, at least partly through the inhibition of the inflammatory phenotype of HSCs.

As the key downstream effector regulating apoptosis, Bcl-2 is inducible in response to LPS/TLR4/NF-κB signaling in activated HSCs, which contributes to cell survival^30^. We also found that LPS promoted the expression of Bcl-2 in HSCs, which exerted a protective effect on cell survival after irradiation. However, miR-146a-5p could efficiently abrogate the LPS/TLR4/NF-kB signaling-mediated anti-apoptotic effects by directly targeting IRAK1 and TRAF6 in HSCs. In the present study, JNK phosphorylation, pro-fibrogenic signals, and α-SMA expression were also found to be up-regulated in HSCs and liver tissues after irradiation and LPS stimulation. These pro-fibrogenic signal and downstream proteins elicited by TLR4 activation were significantly downregulated in the presence of miR-146a-5p. The role of miR-146a-5p in the control of hepatic fibrogenesis may be partly attributed to decreased HSC proliferation, enhanced apoptosis, and reduced production of α-SMA, which indicates the beneficial effects of miR-146a-5p on the regulation of RILD progression. Additionally, we demonstrated that knockdown of TRAF6, but not IRAK1 could reverse irradiation-induced and LPS-induced HSC activation, which was partly suppressed by miR-146a-5p inhibitor. TRAF6 is an important upstream component of the mitogen-activated protein kinases pathway, and silencing of TRAF6 results in the inactivation of ERK, p38 and JNK^31,32^. Especially, JNK plays a potent role in modulating the phosphorylation of Smad2/3 in activated HSCs of CCl4-injured livers^33^. All these results indicate that the NF-κB and JNK pathways, associated with downstream of TLR4 signaling, are involved in miR-14a-5p-mediated regulation of hepatic fibrosis. Although acute injury could activate mechanisms of fibrogenesis in this study, the significant fibrosis may be a progressive response that evolves over weeks to months. Thus, we hypothesize that a longer time period after irradiation and LPS treatment is required for the observation of evident fibrosis, which will be further explored in our future study.

Collectively, our study demonstrates that miR-146a-5p directly inhibits TLR4 signaling to suppress irradiation-induced and endotoxin-induced liver injury as indicated by the reduced production of pro-inflammatory cytokines, suppression of HSC activation, and decreased apoptosis of hepatocytes. Therefore, use of miR-146a-5p to regulate an integrated network of TLR4 pathway may represent a promising approach to prevent RILD.

## Materials and methods

### Cell culture and treatment

The human HSC line LX2 and human normal hepatic cell line LO2 were obtained from Shanghai Advanced Research Institute, Chinese Academy of Sciences. LX2 and LO2 cells were cultured in Dulbecco’s modified Eagle’s medium (DMEM; pH 7.4) supplemented with 10 μg/ml streptomycin sulfate, 100 μg/ml penicillin G, and 10% (v/v) fetal bovine serum (Gibco, Thermo Fisher Scientific, MA, USA). The cells were incubated at 37 °C in a 5% CO2 humidified atmosphere. When the cultures reached confluence, cells were trypsinized and passaged at a ratio of 1:3. Culture media were replaced every 3 days. To avoid the confounding effects of cellular senescence, cells with low passage numbers (<20) were used for the experiments. For irradiation, LX2 cells were treated with a single fraction of 6, 8, or 10 Gy X-ray dose using an ONCOR^TM^ linear accelerator (Siemens, Munich, Germany), with a photon beam energy of 6 MV, dose rate of 3 Gy/min, source surface distance of 100 cm, radiation field size of 10×10 cm^2^ and the gantry at 180°. After a single dose of irradiation, LX2 cells were incubated with different amounts of LPS and then collected at the indicated time points for following experiments.

### miRNA mimics, inhibitors, and siRNA transfection

The miR-146a-5p mimics or inhibitor and their corresponding negative controls were purchased from GenePharma (Shanghai, China) and transfected at a final concentration of 50 nM for mimics and 100 nM for inhibitor in the cells using INTERFERin (Polyplus transfection) according to the manufacturer’s recommendations. For knockdown of IRAK1 and TRAF6, LX2 cells were transfected with IRAK1, TRAF6, or negative control siRNAs using the INTERFERin. The specific siRNA sequences for IRAK1 were: 5′-AGUGGUAGACAUGUAGGAGTT-3′ (sense) and 5′-CUCCUACAUGUCUACCACUTT-3′ (antisense), and for TRAF6 were: 5′-GGGUACAAUACGCCUUACATT-3′ (sense) and 5′-UGUAAGGCGUAUUGUACCCTT-3′ (antisense). The final concentration of target or control siRNA used for transfection was 50 nM.

### Cell proliferation and apoptosis assays

Cell viability was determined from 12 to 120 h by using the Cell Counting Kit-8 (CCK-8, Dojindo,Kumamoto, Japan), according to the manufacturer’s protocol. The optical density (OD) was recorded at 450 nm using a microplate reader. Apoptosis analysis was evaluated by flow cytometry (FCM). After irradiation and LPS stimulation for 72 h, cells were harvested and washed with cold phosphate buffered saline (PBS). Subsequently, the cells were resuspended in binding buffer and stained with the 7-AAD and Annexin V-PE using Annexin V-PE/7-AAD kit (BD Biosciences, MD, USA) according to the manufacturer’s protocol.

### Immunofluorescence staining

LX2 cells or primary HSCs were seeded on glass coverslips. After the indicated treatment, cells were fixed with 4% paraformalde-hyde for 15 min, permeabilized with 0.2% Triton X-100 in PBS for 5 min, and then blocked with 5% bovine serum albumin in PBS for 1 h. After incubation with primary antibodies against NF-κB p65(1:100, NB100-82088, Novus Biologicals, Littleton, USA), α-SMA(1:100, NBP2-33006, Novus Biologicals), or desmin (1:100, NB120-15200, Novus Biologicals) overnight at 4 °C, cells were incubated with Cy3-labeled Goat Anti-Rabbit IgG(1:500, A0516, Beyotime, China) or FITC-labeled Goat Anti-Rabbit IgG (1:500, A0562, Beyotime) for another 1 h. Nuclei were counterstained with 4′, 6-diamidino-2-phenylindole (DAPI, Sigma-Aldrich) and the images were captured using fluorescence microscopy (FV300, Olympus, Japan).

### Hepatocytes co-cultured with supernatants from HSCs

LX2 cells, primary HSCs, LO2 cells, or primary hepatocytes were seeded in a 6-well plate at 2 × 10^6^ cells/well. After altering the expression of miR-146a-5p, IRAK1, or TRAF6, LX2 cells or primary HSCs were treated with 8 Gy X-ray irradiation and 50 ng/ml LPS for 24 h and the supernatants were collected. Meanwhile, the hepatocytes were exposed to a single fraction of 8 Gy X-ray dose and then immediately co-cultured with the supernatants from the above LX2 cells or primary HSCs for 72 h. The morphology of LO2 cells before and after treatment was analyzed by microscopy (FV300, Olympus, Japan). Supernatant levels of ALT and AST were determined using an automatic biochemistry analyzer. Apoptosis of hepatocytes was evaluated by FCM.

### In vivo animal studies

Male C3H/HeN mice (6–7 weeks-old and 16–20 g) were maintained under pathogen-free conditions in the Animal Center of Zhongshan Hospital. All animal experiments were undertaken in accordance with the use of housing facilities for laboratory animal (GB14925-2001) and the protocol was approved by the Animal Ethics Committee of Zhongshan Hospital, Fudan University. The mice were randomized into 6 groups as follow: non-irradiation control, non-irradiation and LPS (0.3 mg/kg) daily intraperitoneal administration, irradiation, irradiation and LPS (0.3 mg/kg) daily intraperitoneal administration, irradiation and LPS (0.3 mg/kg) daily intraperitoneal administration in combination with cholesterol-conjugated miR-146a-5p mimics or negative control via tail vein injection. The whole liver irradiation for mice was conducted according to the method in our previous study^34^. The whole mouse liver was irradiated by helical tomotherapy (HT; Tomo Therapy, Madison,WI, USA) with average administered doses of 30 Gy in a single fraction. For delivery of cholesterol-conjugated miR-146a-5p or negative control (Ribobio, Guangzhou, China), 10 nmol miRNA in 0.2 ml saline buffer was injected through the tail vein once every 3 days for 3 weeks. At 21 days after irradiation, the venous blood of mice was drawn from the eye orbit of mouse before killing. Each serum sample (100 μl) used for LPS detection was quantified by end-point chromogenic limulus amebocyte lysate (LAL) assay (Xiamen BioEndo Technology,Co.Ltd, Xiamen, China) according to the manufacturer’s instructions. Another 100 μl of each serum sample was used for AST and ALT detection using an automatic biochemistry analyzer. After scarification, a part of the liver tissue was harvested from each mouse, fixed in 10% formalin, and embedded in paraffin for histological and immunohistochemistry analysis. Some liver tissues used for hepatic hydroxyproline content detection were analyzed by standard spectrophotometric procedures using Hydroxyproline Colorimetric Assay Kit (BioVision, Milpitas, CA, USA) according to the manufacturer’s protocols. The remaining fresh liver tissues were collected for Quantitative real-time polymerase chain reaction (qRT-PCR) and western blot analysis.

### Isolation of primary hepatocytes and HSCs

The isolation of hepatocytes and HSCs in this study was performed according to previously described methods, with slight modifications^35–37^. Hepatocytes and HSCs were isolated from 9–10 weeks-old male C3H/HeN mice by in situ perfusion with Hank’s solution containing 0.05% collagenase IV. After digestion and filtration, the hepatocytes were obtained from the filtrate after centrifugation at 400 r.p.m. for 5 min at 4 °C. Non-parenchymal liver cells were collected from rest of the filtrate through centrifugation at 2000 r.p.m. for 10 min at 4 °C. After resuspension of the non-parenchymal cells, stellate cells were harvested from the 25% media interface via centrifugation on a discontinuous gradient of Percoll (50 and 25%). Isolated hepatocytes and HSCs were seeded on uncoated plastic culture dishes and cultured in DMEM (pH 7.4) supplemented with 10 μg/mL streptomycin sulfate, 100 μg/mL penicillin G, and 10% (v/v) fetal bovine serum (Gibco). Viability of the isolated cells was determined by a trypan blue exclusion method. The purity of isolated HSCs was assessed by oil red O staining of lipid droplets and double immunofluorescence staining for desmin and α-SMA.

### Quantitative real-time polymerase chain reaction (qRT-PCR)

Total RNA containing miRNA was extracted from cells using TRIzol reagent (Invitrogen) following the manufacturer’s protocol. cDNA was synthesized using the Prime Script RT reagent kit (Takara Bio, Shiga, Japan) and qRT-PCR analysis was performed using SYBR^®^ Premix Ex Taq^™^ (Takara Bio, Japan) in the real-time detection system (ABI7500, USA). The relative expression level of mRNA was normalized to that of endogenous control GAPDH by using 2^−△△Ct^ method. The primer sequences are listed in Supplementary Tables 1 and 2. For miRNA analysis, the first-strand cDNA was synthesized using reverse transcriptase with an miRNA-specific stem-loop primer (RiboBio, Guangzhou, China) and qRT-PCR analysis was conducted using the Bulge-Loop miRNA qRT-PCR Starter Kit (RiboBio, Guangzhou, China). The relative expression level of miRNA was normalized to that of endogenous control U6 by using 2^−△△Ct^ method.

### Western blot

Protein was isolated using RIPA extraction reagent (Pierce Biotechnology, Rockfield, IL,USA) containing phenylmethylsulfonyl fluoride for 30 min at 4 °C. The protein extractions were separated on 10–15% sodium dodecyl sulfate-polyacrylamide gels and transferred onto polyvinylidene difluoride membranes (Immobion-P Transfer Membrane, Millipore Corp., Billerica, MA, USA) blocked with 5% non-fat dry milk, followed by incubation with primary antibodies overnight at 4 °C. Primary antibodies against TLR4 (1:1000, ab183459), IRAK1(1:1000, ab238), TRAF6(1:5000, ab33915), and α-SMA (1:1000, ab32575) were purchased from Abcam (Cambridge, MA, USA). Primary antibodies against NF-κB p65 (1:1000, #8242), phospho-NF-κB p65 (Ser536) (1:1000, #3033), SAPK/JNK (1:1000, #9252), phospho-SAPK/JNK (Thr183/Tyr185) (1:1000, #4668), Smad2 (1:1000, #5339), phospho-Smad2 (Ser465/467) (1:1000, #3101), Bcl-2 (1:1000, #3498), cleaved caspase-3 (Asp175) (1:1000, #9664), cleaved caspase-9 (Asp330) (1:1000, #9501, human specific), and cleaved caspase-9 (Asp353) (1:1000, #9509, mouse specific) were purchased from Cell Signaling Technology (Danvers, MA, USA). Following that, the membrane was incubated with secondary antibody (horseradish peroxidase-labeled IgG anti-rabbit/mouse antibody; Invitrogen, Cambridge, MA, USA) at 1:3000 dilution for 1 h at room temperature. Signals were detected using an ECL system. Anti-GAPDH antibody (1:1000, #2118; Cell Signaling Technology) was used as a loading control.

### Cytokine assays

LX2 cells were transfected with miR-146a-5p inhibitor or mimic, and/or IRAK1, TRAF6, or negative control siRNAs and then stimulated with LPS (50 ng/ml) and/or irradiation (8 Gy) for 24 h. The concentrations of IL-1β, IL-6, and TNF-α in the culture supernatants were measured with ELISA kits (R&D Systems) according to the manufacturer’s instructions. Absorbance was recorded at 450 nm using a microplate reader.

### Immunohistochemistry analysis

Briefly, after de-paraffinization and hydration, the sections were incubated with 3% hydrogen peroxide for 10 min to block endogenous peroxidase activity. Each section was incubated with protein blocker for 15 min using 5% bovine serum albumin at room temperature, followed by incubation with the indicated primary antibodies at their optimal concentration overnight in a wet chamber at 4 °C. After washing with PBS, the sections were incubated with secondary antibody for 1 h at room temperature. The reaction products were developed using diamidobenzidine tetrahydrochloride (DAB) solution (Cell Signaling Technology) for 10 min. The sections were counterstained with hematoxylin. PBS instead of primary antibodies served as a negative control. Immunohistochemical image analysis was performed by software Image Pro Plus (Media Cybernetics, Rockville, MD, USA) with five arbitrarily separated microscopic fields for each slide.

### Statistical analyses

Data were expressed as the mean ± standard error of the mean (S.E.M.) or median with interquartile range. The results were analyzed by Student’s *t*-test, one-way analysis of variance (ANOVA), or Kruskal–Wallis test. Statistical analyses were performed using SPSS 16.0 software (Chicago, IL, USA). *P* *<* 0.05 was considered statistically significant.

## Electronic supplementary material

Supplementary Table 1

Supplementary Table 2

Supplementary Figure 1

Supplementary Figure 2

Supplementary Figure 3

Supplementary Figure Legends
