# Supplementary material for: MicroRNA-146a-5p attenuates irradiation-induced and LPS-induced hepatic stellate cell activation and hepatocyte apoptosis through inhibition of TLR4 pathway
Source: Cell Death Dis. 2018 Jan 18;9(2):22. doi: 10.1038/s41419-017-0038-z (PMC5833436; doi:10.1038/s41419-017-0038-z)
Supplement: Supplementary file 1 — Supplementary Table 1 [file 41419_2017_38_MOESM1_ESM.docx]

**Supplementary Table 1** Primers for LX2

| Gene | sense primer(5'to3') | antisense primer(5'to3') |
| --- | --- | --- |
| TLR4 | AGTTGATCTACCAAGCCTTGAGT | GCTGGTTGTCCCAAAATCACTTT |
| IRAK1 | AGGTTTCGTCACCCAAACATT | CGGGCTGTACCCAGAAGGA |
| TRAF6 | ATGCGGCCATAGGTTCTGC | TCCTCAAGATGTCTCAGTTCCAT |
| Bcl-2 | GGTGGGGTCATGTGTGTGG | CGGTTCAGGTACTCAGTCATCC |
| α-SMA | CTATGAGGGCTATGCCTTGCC | GCTCAGCAGTAGTAACGAAGGA |
| GAPDH | CTGGGCTACACTGAGCACC | AAGTGGTCGTTGAGGGCAATG |
