# Supplementary material for: MicroRNA-146a-5p attenuates irradiation-induced and LPS-induced hepatic stellate cell activation and hepatocyte apoptosis through inhibition of TLR4 pathway
Source: Cell Death Dis. 2018 Jan 18;9(2):22. doi: 10.1038/s41419-017-0038-z (PMC5833436; doi:10.1038/s41419-017-0038-z)
Supplement: Supplementary file 2 — Supplementary Table 2 [file 41419_2017_38_MOESM2_ESM.docx]

**Supplementary Table 2** Primers for mice

| Gene | sense primer(5'to3') | antisense primer(5'to3') |
| --- | --- | --- |
| TLR4 | ATGGCATGGCTTACACCACC | GAGGCCAATTTTGTCTCCACA |
| IRAK1 | TCCTCCACCAAGCAGTCAAG | AAAACCACCCTCTCCAATCCT |
| TRAF6 | AAAGCGAGAGATTCTTTCCCTG | ACTGGGGACAATTCACTAGAGC |
| Bcl-2 | GTCGCTACCGTCGTGACTTC | CAGACATGCACCTACCCAGC |
| α-SMA | GGCACCACTGAACCCTAAGG | ACAATACCAGTTGTACGTCCAGA |
| IL-1β | GAAATGCCACCTTTTGACAGTG | TGGATGCTCTCATCAGGACAG |
| IL-6 | CTGCAAGAGACTTCCATCCAG | AGTGGTATAGACAGGTCTGTTGG |
| TNF-α | TCGTAGCAAACCACCAAGTG | AGATAGCAAATCGGCTGACG |
| GAPDH | TGTGTCCGTCGTGGATCTGA | TTCGTGTTGAAGTCGCAGGAG |
