# Supplementary figures and images for: MicroRNA-146a-5p attenuates irradiation-induced and LPS-induced hepatic stellate cell activation and hepatocyte apoptosis through inhibition of TLR4 pathway
Source: Cell Death Dis. 2018 Jan 18;9(2):22. doi: 10.1038/s41419-017-0038-z (PMC5833436; doi:10.1038/s41419-017-0038-z)

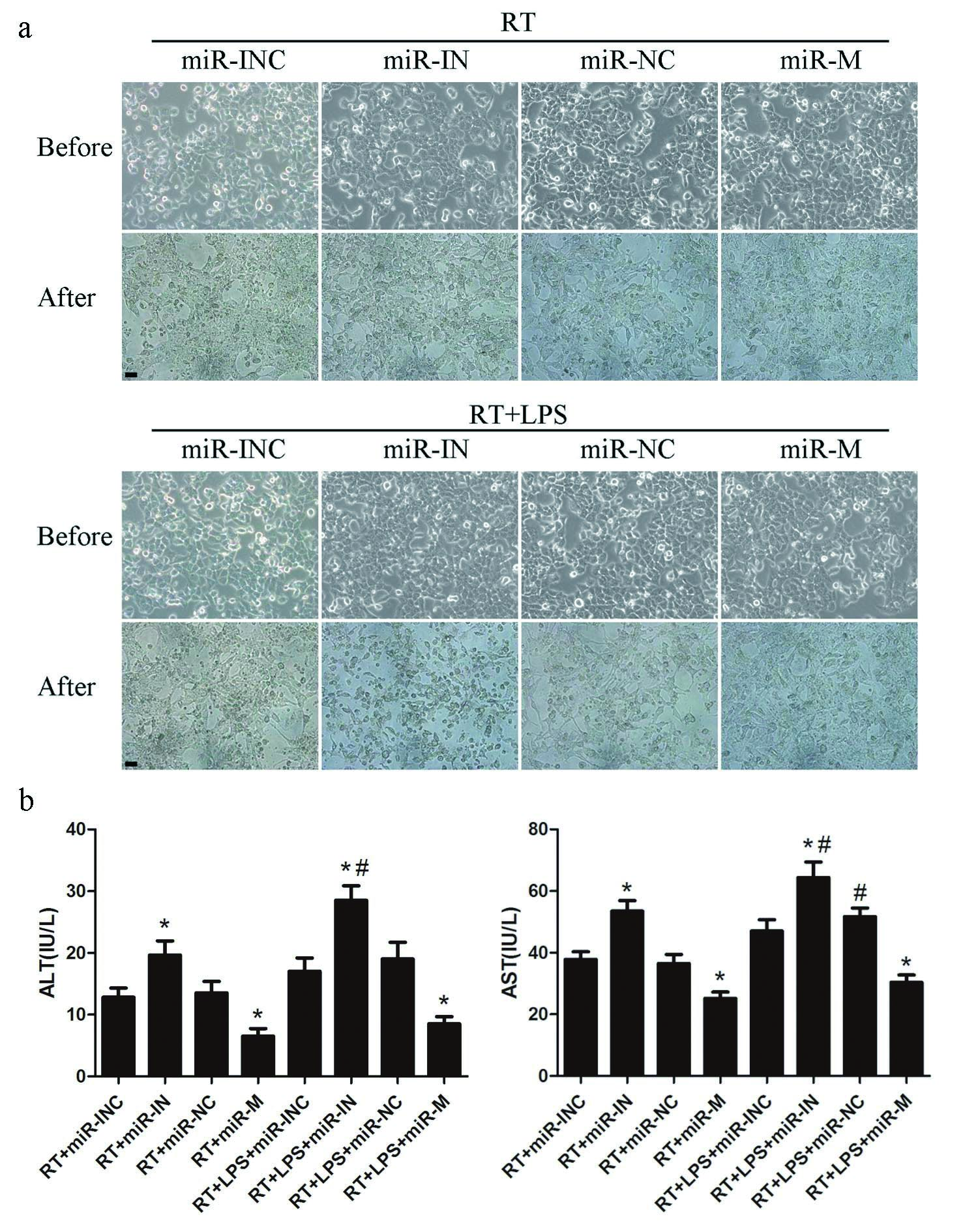

Supplement: Supplementary file 3 — Supplementary Figure 1 [file 41419_2017_38_MOESM3_ESM.jpg]

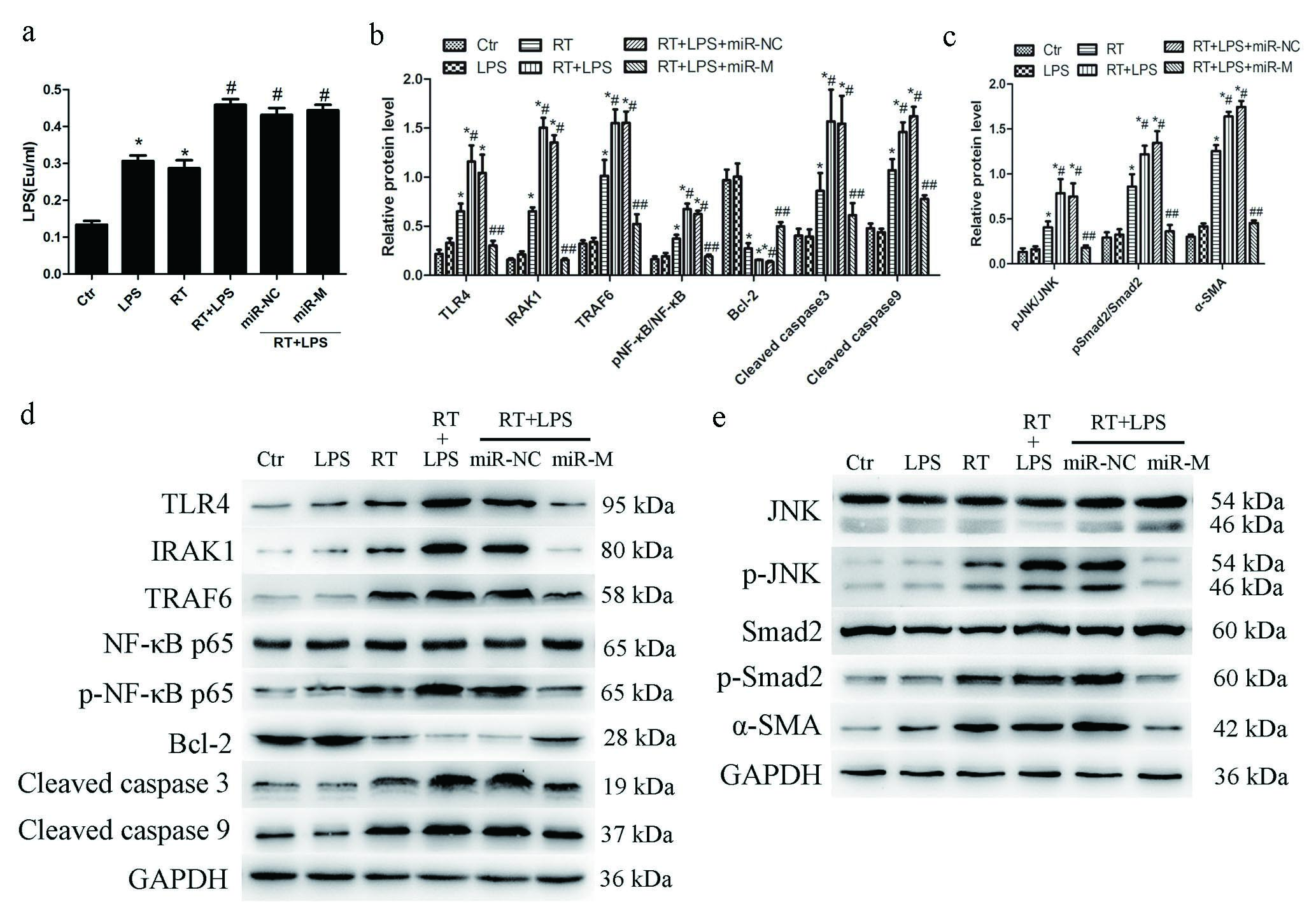

Supplement: Supplementary file 4 — Supplementary Figure 2 [file 41419_2017_38_MOESM4_ESM.jpg]

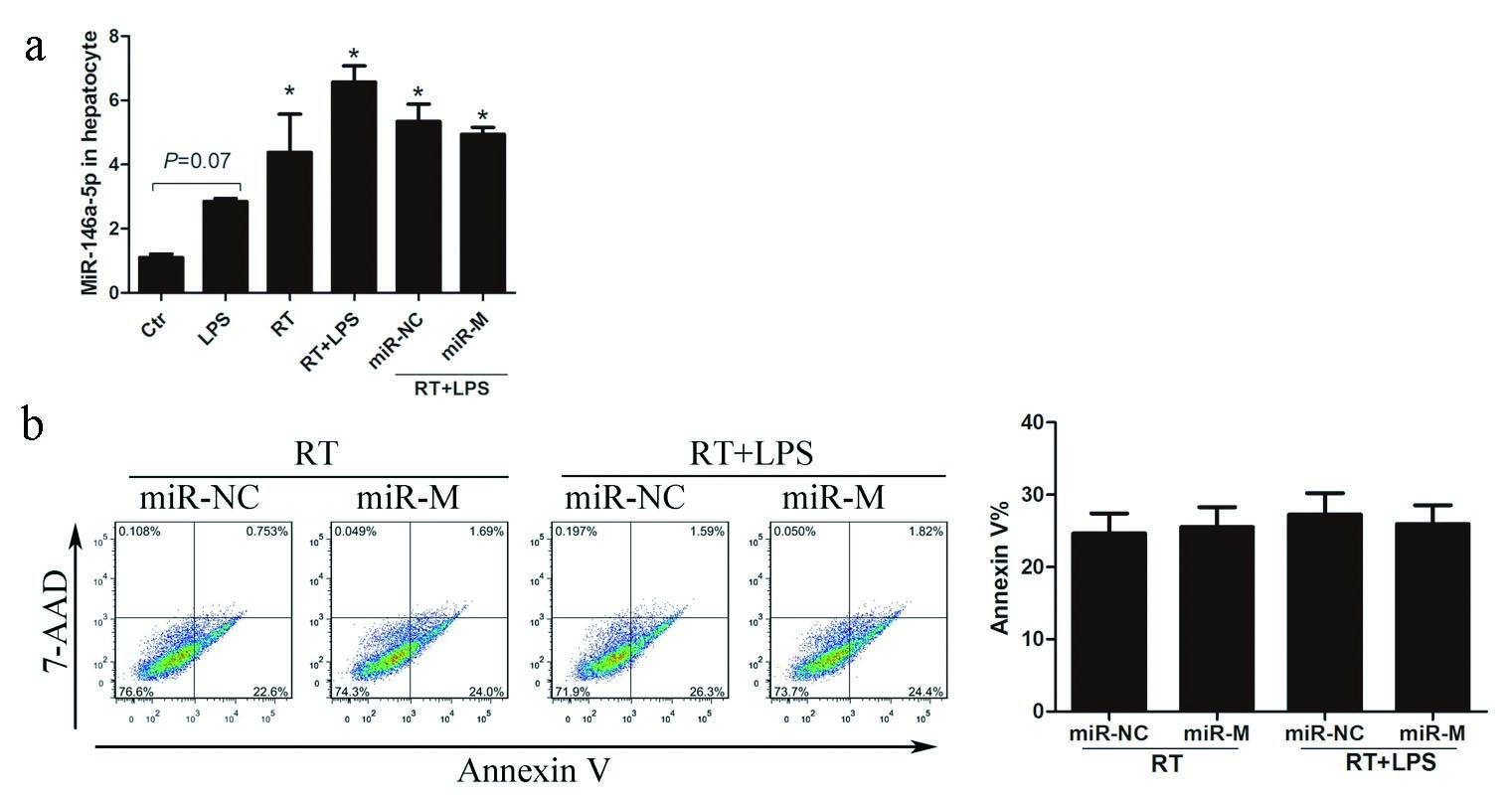

Supplement: Supplementary file 5 — Supplementary Figure 3 [file 41419_2017_38_MOESM5_ESM.jpg]
