# Supplementary material for: MicroRNA-146a-5p attenuates irradiation-induced and LPS-induced hepatic stellate cell activation and hepatocyte apoptosis through inhibition of TLR4 pathway
Source: Cell Death Dis. 2018 Jan 18;9(2):22. doi: 10.1038/s41419-017-0038-z (PMC5833436; doi:10.1038/s41419-017-0038-z)
Supplement: Supplementary file 6 — Supplementary Figure Legends [file 41419_2017_38_MOESM6_ESM.docx]

**MicroRNA-146a-5p attenuates irradiation- and LPS-induced hepatic stellate cell activation and hepatocyte apoptosis through inhibition of TLR4 pathway**

**Supplementary Figure 1** Culture supernatants from irradiated and LPS-stimulated LX2 cells transfected with miR-146a-5p mimics could attenuate irradiation-induced hepatocyte injury. LO2 cells were irradiated and co-cultured with the culture medium from irradiated and LPS-stimulated LX2 cells transfected with miR-146a-5p inhibitors or mimics. (a) Before and 72 h after treatment, the morphology of LO2 cells was analyzed by microscopy. Scale bar: 100 μm. (b) Supernatant levels of ALT and AST in LO2 cells at 72 h after treatments. Data are presented as the mean ± S.E.M. of three independent experiments. **P*<0.05 versus corresponding negative control, #*P*<0.05 versus corresponding RT group. miR-INC, miRNA inhibitors negative control; miR-IN, miRNA inhibitors; miR-NC, miRNA negative control; miR-M, miRNA mimics.

**Supplementary Figure 2** miR-146a-5p negatively regulates TLR4 signaling in the liver of RILD mice. (a) Serum levels of ALT and AST in mice of each group. Data are presented as the mean ± SD (n=6 mice). **P*<0.05 versus Ctr group; #*P*<0.05 versus RT group; ##*P*<0.05 versus miR-NC group. (b and d) Relative protein levels (b) and representative protein bands (d) showing the expression of TLR4, IRAK1 and TRAF6, phosphorylation of NF-κB p65, and the expression of apoptosis-related proteins in the liver tissue of mice in each group. Data are presented as the mean ± S.E.M. of three independent experiments. **P*<0.05 versus Ctr group; #*P*<0.05 versus RT group; ##*P*<0.05 versus miR-NC group. (c and e) Relative protein levels(c) and representative protein bands (e) showing the phosphorylation of JNK and Smad2 and the expression of α-SMA in liver tissue of mice in each group. Data are presented as the mean ± S.E.M. of three independent experiments.**P*<0.05 versus Ctr group; #*P*<0.05 versus RT group; ##*P*<0.05 versus miR-NC group. miR-NC, miRNA negative control; miR-M, miRNA mimics.

**Supplementary Figure 3** miR-146a-5p expression in primary hepatocytes and its influence on hepatocyte apoptosis after irradiation. (a) qRT-PCR analysis showed the expression levels of miR-146a-5p in primary hepatocytes of mice in each group. Data are presented as the mean ± SD (n=3 mice). **P*<0.05 versus Ctr group. (b) Apoptosis analysis of primary hepatocytes transfected with miR-146a-5p mimics or negative control at 72 h after 8 Gy X-ray irradiation and 50ng/ml LPS stimulation.
